# Sensitive Bioanalysis Based on in-Situ Droplet Anodic Stripping Voltammetric Detection of CdS Quantum Dots Label after Enhanced Cathodic Preconcentration

**DOI:** 10.3390/s16091342

**Published:** 2016-08-23

**Authors:** Xiaoli Qin, Linchun Wang, Qingji Xie

**Affiliations:** 1 Key Laboratory of Chemical Biology & Traditional Chinese Medicine Research (Ministry of Education of China), College of Chemistry and Chemical Engineering, Hunan Normal University, Changsha 410081, China; qinxiaoli0413@163.com; 2 Liuzhou Traditional Chinese Medicine Hospital, Liuzhou 545001, China; wllk2000@163.com

**Keywords:** immunoelectrode, aptamer-electrode, screen-printed carbon electrodes, CdS quantum dot-labeled amperometric bioanalysis, signal amplification

## Abstract

We report a protocol of CdS-labeled sandwich-type amperometric bioanalysis with high sensitivity, on the basis of simultaneous chemical-dissolution/cathodic-enrichment of the CdS quantum dot biolabel and anodic stripping voltammetry (ASV) detection of Cd directly on the bioelectrode. We added a microliter droplet of 0.1 M aqueous HNO_3_ to dissolve CdS on the bioelectrode and simultaneously achieved the potentiostatic cathodic preconcentration of Cd by starting the potentiostatic operation before HNO_3_ addition, which can largely increase the ASV signal. Our protocol was used for immunoanalysis and aptamer-based bioanalysis of several proteins, giving limits of detection of 4.5 fg·mL^−1^ for human immunoglobulin G, 3.0 fg·mL^−1^ for human carcinoembryonic antigen (CEA), 4.9 fg·mL^−1^ for human *α*-fetoprotein (AFP), and 0.9 fM for thrombin, which are better than many reported results. The simultaneous and sensitive analysis of CEA and AFP at two screen-printed carbon electrodes was also conducted by our protocol.

## 1. Introduction

Bioanalysis on the basis of a variety of bioaffinity events that are naturally of high specificity has attracted great academic and industrial attention [1,2,3]. Improving the bioanalysis sensitivity is widely concerned in many areas including biomedical and environmental analysis [4,5,6]. Various biolabeling strategies are frequently used in bioaffinity-based bioanalysis, because the bioaffinity events usually fail to directly give great analytical signals [7,8,9]. Two kinds of biolabeling protocols have been reported for the bioaffinity-based bioanalysis to date, i.e., molecule-level biolabeling (e.g., radioactive labeling [10,11] and enzyme labeling [12,13]) and nanometer-level biolabeling [14,15]. Many nanomaterials, e.g., gold nanoparticles (AuNPs), silver nanoparticles, metal sulphide/selenide/telluride quantum dots (QDs), graphene, and carbon nanotubes, can be used as biolabels to output and amplify the analytical signals, mainly owing to the unique optical, electronic, electrochemical, catalytic, and/or mechanical properties of nanomaterials [15,16,17].

Various optical and electrochemical methods as well as their hyphenation with chromatographic or magnetic separation have been widely employed in bioanalysis [18,19]. Electrochemical methods have been intensively explored for rapid bioanalysis due to the high sensitivity and selectivity, low limits of detection (LODs), facile operation, simple instrumentation, and scope for miniaturization [20,21,22]. The metal-labeled amperometric bioanalysis (MLAB) method involving a sandwich-type bioaffinity interface has been proven promising, which accesses the amperometric signal of metal biolabels either by their chemical dissolution, transfer of the lysate into another electrolyte for cathodic enrichment of the atomic metal, and then anodic stripping voltammetry (ASV) analysis, or by in situ amperometric analysis directly at the bioelectrode without the metal-enrichment step [23,24,25,26,27]. However, signal mining from the metal labels in the two protocols is somewhat limited due to either the solution-dilution effect or the intrinsically short distance of electron communication [28,29]. Obviously, amplification of MLAB signals is very interesting.

**Scheme 1:**
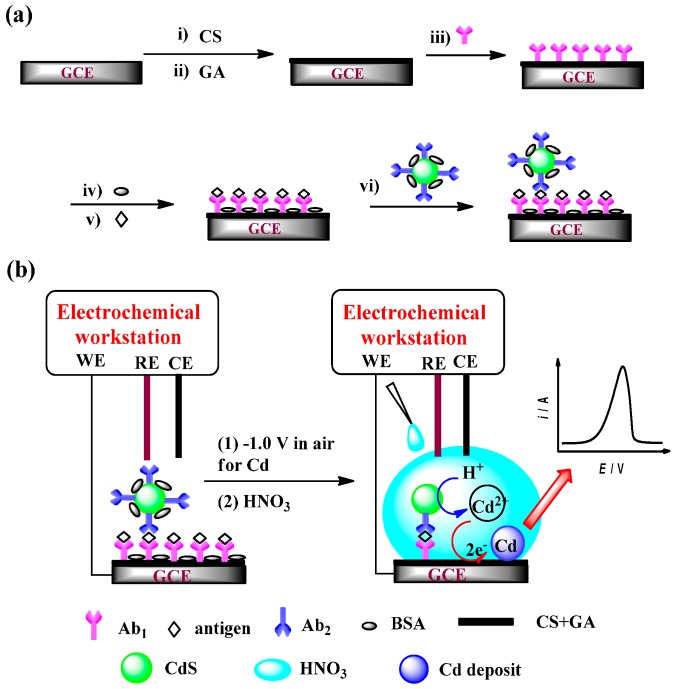
Steps for preparing the immunosensor (**a**) and outputting the ASV signal (**b**).

Herein, we report a CdS-labeled MLAB protocol for sandwich-type immunoanalysis and aptamer-based bioanalysis, on the basis of simultaneous chemical-dissolution/cathodic-enrichment of the CdS quantum dots biolabel and then in-situ ASV analysis directly on the bioelectrode. Major steps of our protocol are depicted in Scheme 1 (here, immunoassay, as an example). First, the primary antibody (Ab_1_) was covalently immobilized on a chitosan (CS) modified glassy carbon electrode (GCE) by glutaraldehyde (GA) crosslinking, bovine serum albumin (BSA) was used to block the possible remaining active sites against nonspecific adsorption, and then the target antigen was immunologically immobilized. The second antibody (Ab_2_) labeled with CdS QDs (Ab_2_-CdS) was then captured on the electrode, followed by thorough water-rinse and nitrogen-drying. Second, 5 μL of 0.1 M aqueous HNO_3_ was added to dissolve the CdS label and connect the three-electrode electrolytic cell for diffusion-controlled potentiostatic cathodic preconcentration of metallic Cd (−1.0 V vs. SCE). Note that the potentiostatic operation was started before the HNO_3_ addition (safe in the potentiostatic mode), so as to minimize the diffusion-layer thickness to capture Cd as entirely as possible from the CdS QDs biolabel, as proven by our recent efforts [30,31,32]. Finally, differential pulse ASV analysis of Cd was conducted to quantify the antigen analyte. Our protocol has been used for sandwich-type immunoanalysis and aptamer-based bioanalysis of several proteins, giving limits of detection (LODs) of 4.5 fg·mL^−1^ for human immunoglobulin G (IgG), 3.0 fg·mL^−1^ for human carcinoembryonic antigen (CEA), 4.9 fg·mL^−1^ for human *α*-fetoprotein (AFP), and 0.9 fM for thrombin with the CdS QDs label, which are better than many reported results (Table 1).

## 2. Materials and Methods

### 2.1. Apparatus and Materials

All electrochemical immunoassays were performed on a CHI660A electrochemical workstation or a CHI1040B multichannel potentiostat (Chenhua Instruments Co., Shanghai, China). The CHI1040B multichannel potentiostat can work with eight independent three electrode cells or eight working electrodes (WEs) in the same solution with common reference electrode (RE) and counter electrode (CE). A disk GCE with 3.0 mm diameter and a platinum wire with 0.1 mm diameter (Chenhua Instruments Co.) served as the WE and the CE, respectively. A KCl-saturated calomel electrode (SCE) of a small-sized salt bridge filled with saturated KNO_3_ served as the RE. All potentials here are cited versus SCE, unless otherwise specified. The screen-printed carbon electrodes (SPCEs, area = 2 mm^2^) was made by an electric flat screen printer (AT-25PA, ATMA Tong Yuan M/C Ind. Co., Ltd., Kunshan, China).

IgG and goat anti-IgG (anti-IgG) were purchased from Beijing Dingguo Changsheng Biotechnology Co., Ltd., Beijing, China. Monoclonal mouse anti-CEA (anti-CEA), CEA, anti-AFP, and AFP were purchased from Beijing Key Biotech. Co., Ltd., Beijing, China. 1-Ethyl-3-(3-dimethylaminopropyl) carbodiimide hydrochloride (EDC), *N*-hydroxysuccinimide (NHS), human *α*-thrombin, and BSA were purchased from Sigma-Aldrich Chemical Co. (St. Louis, MO, USA). CS from crab shells (90% deacetylated) was commercially obtained from Sinopharm Chemicals Co., Ltd. (Shanghai, China). GA (25% aqueous solution) was purchased from Alfa Aesar China Ltd. (Tianjin, China). The washing and blocking buffer for immunoassay was 0.01 M phosphate buffer (10 mM NaH_2_PO_4_ − Na_2_HPO_4_ + 0.15 M NaCl, pH 7.4). Fifty millimoles of Tris-HCl buffer containing 100 mM NaCl, 5 mM KCl, 1 mM MgCl_2_, 5 mM CaCl_2_ (pH 7.4) was used for construction and rinse of the aptamer-electrode. 0.25 wt% CS solution was prepared in 0.10 M acetate buffer (pH 5.4). All other chemicals were of analytical grade or better quality. Milli-Q ultrapure water (Millipore, ≥18 MΩ·cm) was used in all experiments. The clinical serum samples were approved by the Ethical Committee of the Liuzhou Traditional Chinese Medicine Hospital, Guangxi Zhuang Autonomous Region, China, and the CEA and AFP levels had been analyzed by chemiluminescence in the hospital. Written informed consent was obtained from all donors. The nucleic acid aptamers with the following sequences were purchased from Sangon biotech Co., Ltd., Shanghai, China.

Aptamer-I (Apt_1-NH2_): 5′-NH_2_-(CH_2_)_6_-T_10_GGTTGGTGTGGTTGG-3′

Aptamer-II (Apt_2_): 5′-(TC)_10_AGTCCGTGGTAGGGCAGGTTGGGGTGACT-3′

### 2.2. Preparation of Ab_2_-CdS or Apt_2_-CdS Conjugates

Prior to use, all glassware was thoroughly cleaned in aqua regia (*V*_HNO3_:*V*_HCl_ = 1:3), rinsed with ultrapure water, and oven-dried. The CdS QDs of (30 ± 5) nm diameter were prepared as reported previously [54]. Briefly, in a 100 mL Erlenmeyer flask, 10 mL of 0.01 M Cd(CH_3_COO)_2_ and 5 mL of 0.01 M CH_3_CSNH_2_ were mixed, with slowly added 0.3 mL of 0.1 M sodium hexametaphosphate solution as the stabilizer. The solution was adjusted to pH 9.5 with 0.1 M NaOH and vigorously stirred to allow reaction for about 1 h, finally yielding a yellowish sol of CdS QDs. 2.5 μL of 0.7 mg·L^−1^ cysteine solution was added to the sol, in a volumetric flask to allow reaction for 24 h. Then, 1.5 mL of the cysteine-functionalized CdS nanoparticles dispersion was mixed with fresh prepared 50 μL 0.1 M EDC-HCl and 50 μL 0.1 M NHS. The samples were incubated at room temperature for 1 h under shaking, and washed thoroughly with buffer to remove excess EDC-HCl and NHS. Next, 10 μg antibody or 1 nmol Apt_2_ was added and gently mixed at 4 °C for 20 h. The supernatant was discarded after centrifugation at 4800 rpm for 30 min, then the soft sediment was washed with 0.01 M phosphate buffer (pH 7.4). Repeating the centrifugation, the CdS conjugates were finally redispersed in 0.5 mL 0.01 M pH 7.4 phosphate buffer containing 1.0% (*w*/*v*) BSA or 100 μM BSA and stored at 4 °C prior to use.

### 2.3. Preparation of Immunoelectrodes

GCE was carefully polished with aqueous alumina slurries (particle size of 0.5 μm and then 0.05 μm). After water rinse, the polished GCE was ultrasonically washed in water, ethanol, and water for 5 min each to eliminate residual alumina powder. The GCE was treated with concentrated sulfuric acid for 15 s and then water-rinsed. Cyclic voltammertry (CV) from −1.0 to 1.0 V at 100 mV·s^−1^ was performed in 0.50 M aqueous H_2_SO_4_ until CV curves became reproducible. The cleaned GCE was used for immobilization of antibody.

First, 2.5 μL of 0.25 mg·mL^−1^ CS was dropped and dried at room temperature on the WE, followed by activating with 2.5% GA (in 50 mM pH 7.4 phosphate buffer) for 2 h and washing with water (GA-CS/GCE). Afterward, 6 μL of 1 mg·mL^−1^ Ab_1_ was dropped on the WE and incubated at room temperature for 1 h and then at 4 °C overnight in a moisture-saturated environment. Subsequently, excess Ab_1_ was removed with the washing buffer. A 1-h treatment with BSA (3%, 6 μL) blocking solution was applied to the nonspecific sites, followed by washing with buffer.

The assay of antigen is shown in Scheme 1. The immunoelectrode was first incubated with 6 μL of antigen standard solution or serum sample at 37 °C for 60 min. After rinsed with washing buffer, the immunoelectrode was incubated at 37 °C in phosphate buffer containing Ab_2_-CdS for 40 min. After, the immunoelectrode was rinsed thoroughly with phosphate buffer and ultrapure water to remove the nonspecifically-bound species, and the final immunoelectrode Ab_2_-CdS/antigen/BSA/Ab_1_/GA-CS/GCE was obtained.

**Scheme 2:**
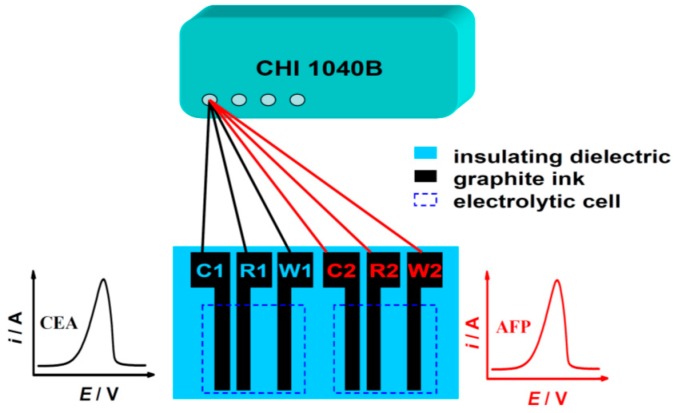
Schematic diagram for simultaneous two-analyte immunoassay (not to scale). Here, W1, R1, and C1 denote WE1, RE1, CE1, and W2, R2, and C2 denote WE2, RE2, and CE2, respectively.

The two-analyte immunoassay using CdS QDs as biolabels was similarly conducted. The SPCEs containing two graphite WEs, two graphite quasi-references, and two graphite auxiliary electrodes were prepared by the aforementioned screen-printing equipment, as shown in Scheme 2. Two electrochemical microcells were constructed by the insulating layer printed around the working areas. The two-analyte immunoelectrodes using the working SPCEs were constructed similarly to those using GCEs as before.

### 2.4. Preparation of Aptamer-Electrodes

10 μL of 1 μM Apt_1-NH2_ was added to the GA-CS/GCE surface, followed by incubation at room temperature for 1 h and at 4 °C overnight in a moisture-saturated environment. Excess Apt_1-NH2_ was removed with the washing buffer, and a 1-h treatment with BSA (100 μM, 10 μL) blocking solution was applied to block the nonspecific sites, followed by buffer washing. The electrode was incubated at 37 °C for 2 h with 10 μL buffer containing thrombin at different concentrations. After careful washing with the buffer to remove non-captured thrombin, 10 μL of Apt_2_-CdS was dropped to the WE and incubated at 37 °C for 2 h. The electrode was then rinsed with ultrapure water three times to obtain an aptamer-electrode of Apt_2_-CdS/thrombin/Apt_1-NH2_/GA-CS/GCE, which was stored in a dry environment before use.

### 2.5. Conventional Cell Measurement Procedures

We switched on the potentiostat at −1.0 V in air, so as to ensure the synchronization of diffusion-controlled Cd electrodeposition soon after the WE, RE, and CE were connected by the added electrolyte solution. The CdS marker was dissolved by the addition of 5 μL 0.1 M HNO_3_ solution for simultaneous cathodic preconcentration of atomic Cd. During 500 s preconcentration, 8.5 mL of 0.3 M aqueous sodium acetate (NaAc) was added at the last 50 s to regulate the pH to about 5. Differential pulse ASV from −1.0 to −0.45 V, with 4 mV potential steps, 50 mV amplitude, and 50 ms pulse width, was performed to record the ASV currents. For the conventional solution-replacement protocol, the solution of dissolved Cd^2+^ ions (5 μL) was transferred into 995 μL of 0.2 M acetate buffer at pH 5.2 as the electrolyte solution, and ASV analysis was conducted on another cleaned GCE (−1.0 V).

### 2.6. SPCE Measurements

The two-analyte immunoassay, which should intrinsically have a time-efficiency higher than the separate one-analyte immunoassays (sharing the same time of cathodic preconcentration), was conducted using the CHI1040B electrochemical workstation (Scheme 2). Briefly, −1.0 V vs. graphite reference were first applied to each of the two electrolytic cells in Scheme 2 in air, which can allow diffusion-controlled Cd electrodeposition in the solution. Then, we simultaneously added two independent liquid drops (each 5 μL 0.1 M HNO_3_) to dissolve the CdS biolabel and preconcentrate atomic Cd for 500 s. Differential pulse ASV with the same parameters as before was performed.

## 3. Results

### 3.1. Simulated Experiments for Evaluating the Signaling Efficiency of our Protocol

We conducted the following simulated experiments to compare the signaling efficiency (*δ*, defined as in Equation (1)) of our protocol with those of conventional ones. An appropriate amount of CdS QDs (*n*_CdS-cast_ in mol) were cast-coated on a bare GCE and dried in air, and anodic stripping linear sweep voltammetry (LSV) was used to detect the amount of cast-coated CdS (*n*_CdS-LSV_ in mol).

(1) $$\delta =n_{\text{CdS-LSV}}/n_{\text{CdS-cast}}=Q/(zFn_{\text{CdS-cast}})$$

where *F* is the Faraday constant (96485.3 C·mol^−1^), and *z* is the number of electrons transferred (*z* = 2 here).

**Figure 1:**
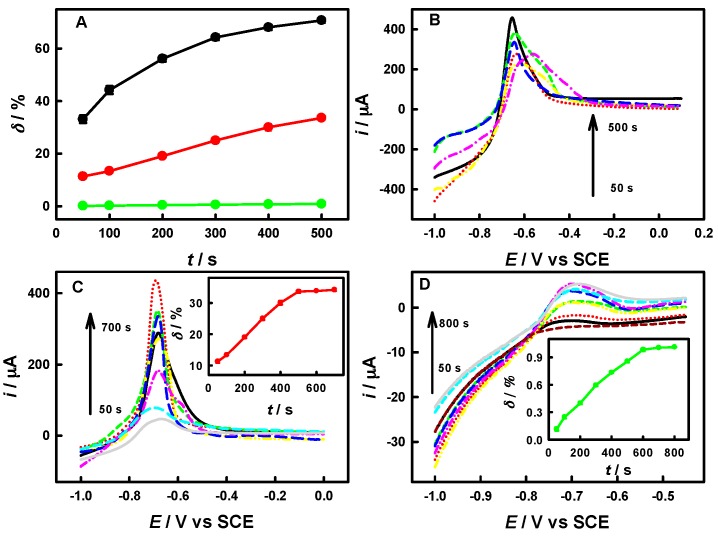
*δ* versus preconcentration-time curves (**A**, and Insets of **C** and **D**, *n* = 3) and anodic stripping LSV curves of the CdS QDs cast-coated on GCE (B, C and D, 100 mV·s^−1^) for our protocol (**B**), the similar protocol but without the beforehand exertion of a cathodic potential in air (**C**), and conventional solution-replacement protocol (**D**). Ten microliters of 10-fold diluted 6.5 mM CdS QDs dispersion was used for cast coating.

The anodic stripping LSV curves and *δ* as functions of cathodic-enrichment time are shown in Figure 1. The maximum *δ* for our protocol was as high as 70.7%, after dissolution of CdS QDs and 500-s cathodic enrichment in 5 μL of 0.1 M HNO_3_ and then LSV stripping. In contrast, the similar protocol but the cathodic-concentration potential was applied after HNO_3_ addition gave *δ* = 34.2% for 500-s preconcentration, highlighting the importance of the beforehand infliction of a cathodic potential in air in our protocol, since the WE in our protocol can more efficiently capture Cd by cathodic reduction of nearby Cd^2+^ on the WE immediately after the acidic dissolution of CdS. In addition, the *δ* even for 600-s enrichment was only 1.0% for the conventional solution-replacement protocol by dissolving the CdS QDs with 5 μL of 0.1 M HNO_3_ and then transferring it into 995-μL 0.10 M acetate buffer (pH 5.2) for enrichment at −1.0 V and LSV stripping. The above results simply for cast-coated CdS QDs confirm the maximum signaling efficiency of our protocol versus conventional ones.

The volume of 0.1 M HNO_3_ used to dissolve CdS QDs was optimized. As shown in Figure S1, *δ* decreased with the increase of HNO_3_ volume, because a smaller volume of HNO_3_ solution can lead to a thinner diffusion-layer for enhanced enrichment of atomic Cd on the WE and, thus, a larger ASV peak. Hence, 5-μL HNO_3_ will be used below to maximize the signal.

### 3.2. Immunoassay of IgG, CEA and AFP

**Figure 2:**
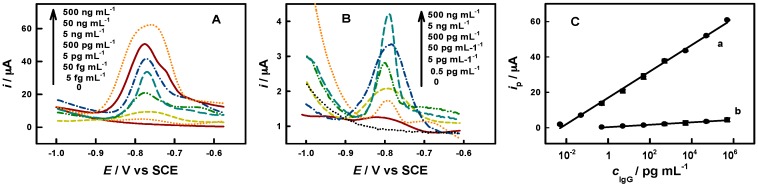
Differential pulse ASV curves for IgG immunoassay (**A**,**B**) and corresponding calibration curves (**C**, *n* = 3). Panel A and curve a in panel C are for our protocol. Panel B and curve b in panel C are for the conventional solution-replacement protocol.

Our protocol can be well used for immunoassay of proteins. Under optimized conditions, the stripping peak current is linear with the common logarithm of IgG concentration from 5 fg·mL^−1^ to 500 ng·mL^−1^, with a sensitivity of 7.5 μA·dec^−1^ (dec means decade) and a LOD of 4.5 fg·mL^−1^ (*S*/*N* = 3), as shown in Figure 2. The LOD is much better than that experimentally obtained from the conventional solution-replacement protocol (0.4 pg·mL^−1^, Figure 2) and many literature-reported values (Table 1). Three repetitive measurements of 0.500, 5.00, and 50.0 ng·mL^−1^ IgG yielded reproducible ASV signals with relative standard deviations (RSDs) of (6% ± 2%), indicating acceptable reproducibility.

**Figure 3:**
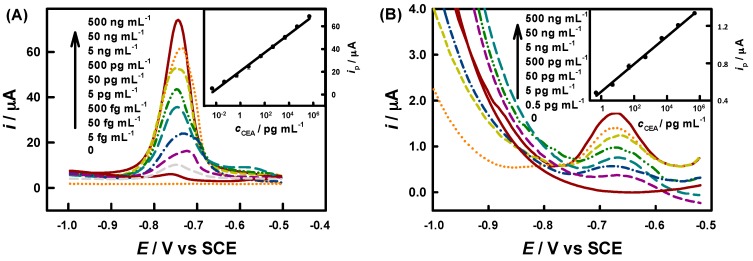
Differential pulse ASV curves for CEA immunoassay (**A**) for our protocol and (**B**) for the conventional protocol) and corresponding calibration curves (inset, *n* = 3).

**Figure 4:**
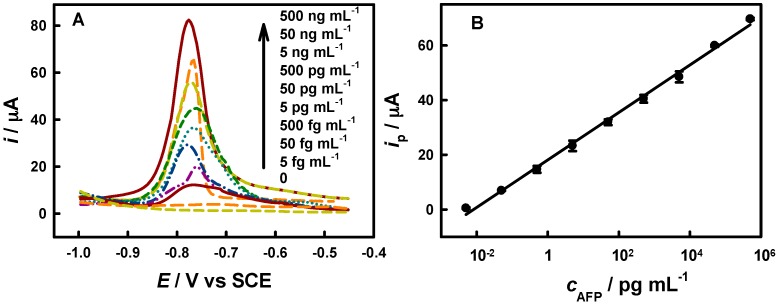
Differential pulse ASV curves for AFP immunoassay (**A**) and corresponding calibration curves (**B**) by our protocol (*n* = 3).

CEA is a very important clinical diagnosis biomarker for a wide range of malignancies, such as breast cancer, colorectal cancer, and gastric cancer, and is usually immunologically determined [45,46]. Our protocol was also used to detect CEA (Figure 3A). Under optimized conditions, the stripping peak current is linear with the common logarithm of CEA concentration from 5 fg·mL^−1^ to 500 ng·mL^−1^, with a sensitivity of 8.1 μA·dec^−1^ and a LOD of 3.0 fg·mL^−1^ (*S*/*N* = 3). The LOD is much better than that experimentally obtained from the conventional solution-replacement protocol (0.2 pg·mL^−1^ for CdS QDs label, Figure 3B) and the literature-reported values (Table 1). Similarly, our protocol also gave much enhanced analytical performance for immunoassay of AFP (LOD = 4.9 fg·mL^−1^), as shown in Figure 4 and Table 1.

To evaluate the analytical reliability and application potential of our protocol, CEA in human-serum samples was determined by our method (only 6 μL sample required for each test) for comparison with the reference results of chemiluminescence assay. As listed in Table S1, our results agree well with the reference results with relative deviations within ±7%, proving the high analytical reliability and application potential of our protocol. The storage stability of the prepared immunoelectrodes was examined by keeping them under dry conditions at 4 °C, and both IgG and CEA gave over 90% of the initial responses after two weeks.

### 3.3. Aptamer-Based Bioanalysis

**Figure 5:**
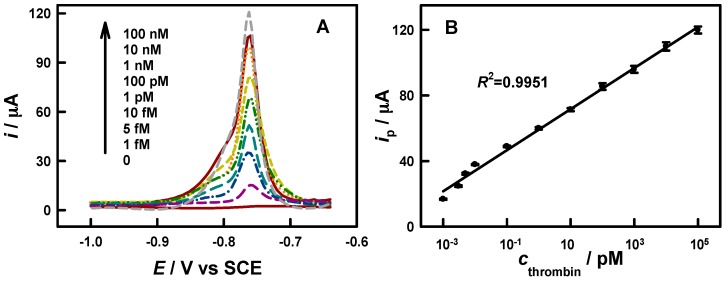
Differential pulse ASV curves for thrombin aptamer-electrodes (**A**) and corresponding calibration curves (**B**) using our protocol (*n* = 3).

**Figure 6:**
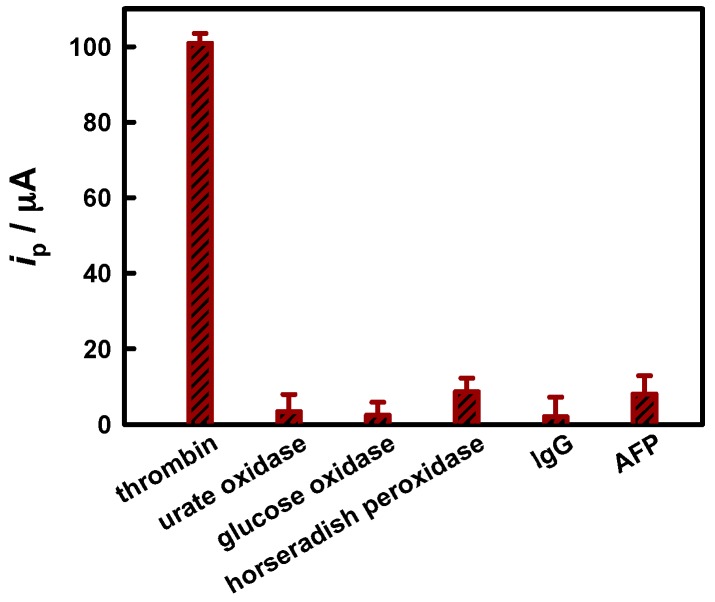
The responses of the prepared aptamer-electrode to 10 nM thrombin and several other proteins each at 1 μM.

Our protocol also can be well used for aptamer-based bioanalysis. Aptamers are nucleic acids that can specifically bind to their targets [55,56,57]. Here, thrombin and a pair of its aptamers were used as a model system. Under the optimum conditions, the stripping peak current is linear with the common logarithm of thrombin concentration from 1 fM to 10 nM, with a sensitivity of 12.5 μA·dec^−1^ and a LOD of 0.9 fM (*S*/*N* = 3), as shown in Figure 5. The LOD is much lower than the previously-reported results (Table 1). We also investigated the selectivity and reproducibility of our protocol. All of the responses to five nonspecific proteins, each at a 100-fold concentration of thrombin, were less than 9% of that to thrombin, as shown in Figure 6. One and 5 nM standard thrombin aqueous solutions were detected by five bioelectrodes, and the RSDs are 8.7% and 7.3%, respectively. As listed in Table 2, our method was evaluated in human blood serum substrate by the standard addition method. The recovery and RSD are acceptable, indicating the application potential of our method for analysis of complex biological samples.

### 3.4. Simultaneous Two-Target Immunoassay

**Figure 7:**
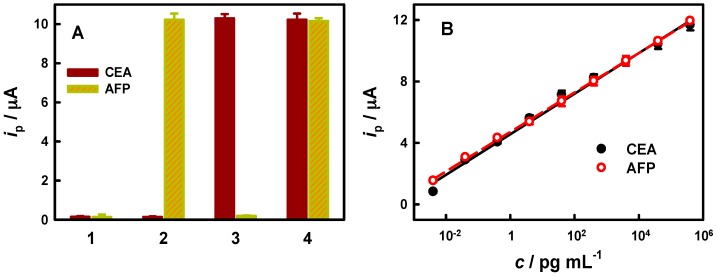
(**A**) Differential pulse ASV responses at a BSA/anti-CEA/SPCE and a neighboring BSA/anti-CEA/SPCE after incubated with blank control (1), 40 ng·mL^−1^ AFP (2), 40 ng·mL^−1^ CEA (3), 40 ng·mL^−1^ of AFP and CEA (4), and then Ab_2_-CdS QDs for immunoassay by our protocol; and (**B**) calibration curves for simultaneous analysis of CEA and AFP.

Our protocol was used for simultaneous two-target immunoassay at two SPCEs (Scheme 2). Here, the two electrolytic cells were independent, since we used two independent liquid drops, each with 5-μL volume. This design can exclude the crosstalk generally resulting from the diffusion of electroactive species on one electrode to neighboring electrodes, as confirmed in Figure S2 and Figure 7. We prepared BSA/anti-CEA/SPCE and BSA/anti-AFP/SPCE on the chip, then incubated with blank solution, 40 ng·mL^−1^ CEA, or/and 40 ng·mL^−1^ AFP, and then Ab_2_-CdS QDs for immunoassay by our protocol. As expected, anodic stripping responses were observable only on the immunoelectrodes with corresponding capture antibodies (Figure 7A), excluding the cross-reactivity between the two analytical systems. Here, the simultaneous two-target immunoassay gave linear response ranges from 4 fg·mL^−1^ to 400 ng·mL^−1^ with a sensitivity of 1.32 μA·dec^−1^ and a LOD of 2.8 fg·mL^−1^ (*S*/*N* = 3) for CEA, as well as from 4 fg·mL^−1^ to 400 ng·mL^−1^ with a sensitivity of 1.28 μA·dec^−1^ and a LOD of 3.0 fg·mL^−1^ (*S*/*N* = 3) for AFP, respectively, as shown in Figure 7B. The analytical performance different from those at GCE-based bioelectrodes comes from the different electroactivity of the GCE and SPCE substrates. As listed in Table 3, our two-target immunoassay protocol was used for simultaneous CEA and AFP assay in clinical human-serum samples. No significant differences were obtained between our results and the hospital results (within ±7% relative deviation (RD)). Thus, we believe that our protocol can be used for simultaneous multianalyte bioanalysis of practical samples on disposable biochips.

## 4. Conclusions

In conclusion, we have demonstrated a biosensing protocol on the basis of CdS quantum dot biolabeling and in situ droplet ASV detection with enhanced cathodic preconcentration, which enables bioassay of CEA, AFP, and thrombin at fg·mL^−1^ or fM levels, and performs better than many reported analogues. Immunoassays of CEA and AFP in clinical samples by our protocol gave results agreeable with the hospital results. Our protocol has the advantages of high sensitivity, wide linear detection range (ca. six orders of magnitude), low LOD, good accuracy/precision/stability, easy operation, and small consumption of reagents/samples. Compared with our recent efforts [30,31,32], the CdS biolabeling here is more convenient in acidic dissolution and ASV detection, though further increasing the sensitivity and lowering the LOD rely on additional signal-amplification protocols. The proven capability of simultaneous two-target immunoassay may be extended for simultaneous multianalyte detection on high-throughput disposable biochips (bioelectronic coding [1,5] also possible), which holds great potential for disease diagnosis and other biosensing applications.

## Figures and Tables

**Table 1:** Comparison of some sandwich-type bioelectrodes for bioassay *.

Analyte	Label	Analytical Technique	LDR/ng·mL^−1^	LOD/ng·mL^−1^	Ref.
IgG	FITC	CRET	0.03–0.6	4.35 × 10^−3^	[33]
	CdS QDs	Photoelectrochemical	5 × 10^−4^–5	5 × 10^−4^	[15]
	Glucose	Chronoamperometry	0.005–1	0.002	[34]
	CdTe QDs	Fluorometry/SWV	0.1–500/5 × 10^−3^−100	0.03/0.005	[35]
	AuNPs	ASV for Au(III)	0.5–100	0.5	[36]
	AuNPs and ALP	ASV for catalytically-deposited Ag	0.01–250	4.8 × 10^−3^ 6.1 × 10^−3^	[37]
	CdS QDs	Differential pulse ASV	5 × 10^−6^–500	4.5 × 10^−6^	This work
AFP	CNSs-HRP	SWV	0.05–6	0.02	[38]
	CdTe-GOx	Photoelectrochemistry	5 × 10^−4^–1 × 10^4^	1.3 × 10^−4^	[39]
	Au-MNCs	Dynamic light scattering	0.01–50	0.01	[40]
	PLNPs	FRET	0.8–45	0.41	[41]
	Label-free	Electrochemiluminescence	1 × 10^−4^–10, 10–320	1 × 10^−4^	[42]
	Label-free	Differential pulse ASV	0.5–50	0.1	[43]
	CdS QDs	Differential pulse ASV	5 × 10^−6^–500	4.9 × 10^−6^	This work
CEA	Label-free	Differential pulse ASV	0.5–80	0.05	[43]
	AuNPs	Differential pulse ASV	1 × 10^−5^–100	3.0 × 10^−6^	[44]
	Cy3	Fluorescence	0.3–100	0.09	[45]
	ALP	Chemiluminescence	1–120	0.6	[46]
	Pt–Ag alloy	Electrogenerated chemiluminescence	1 × 10^−5^–10	3.0 × 10^−6^	[47]
	Label-free	Differential pulse ASV	0.5–45	0.2	[48]
	CdS QDs	Differential pulse ASV	5 × 10^−6^–500	3.0 × 10^−6^	This work
Thrombin	QDs	SWV	0.02–0.5	0.02	[1]
	Fe_3_O4@CdSe	Electrochemiluminescence	1 × 10^−3^–5.0 nM	0.12 pM	[49]
	AuNPs	Colorimetric detection	0.115–3.7 pM	14 fM	[50]
	AuNPs	Absorption spectra for catalytically deposited Au	2–167 nM	2 nM	[51]
	Label-free	EIS	0.12–30 nM	0.06 nM	[52]
	AuNPs	SPR	0.1–75 nM	0.1 nM	[53]
	CdS QDs	Differential pulse ASV	1 × 10^−6^–10 nM	0.9 fM	This work

* Immunoassay for AFP and CEA, and aptasensing for thrombin. FITC: fluorescein isothiocyanate; CRET: chemiluminescence resonance energy transfer; SWV: square wave voltammograms; ALP: alkaline phosphatase; CNSs: carbon nanospheres; MNCs: magnetic nanoparticle clusters; PLNPs: persistent-luminescence nanoparticles; FRET: fluorescence resonance energy transfer; EIS: electrochemical impedance spectroscopy; SPR: surface plasmon resonance. LDR and LOD for thrombin in molar concentrations are separately given in corresponding rows.

**Table 2:** Detection of thrombin in the human blood serum substrate (*n* = 3) by our method. The sera were 10-fold diluted with Tris-HCl buffer (pH = 7.4).

Added/nmol·L^−1^	Measured/nmol·L^−1^	RSD/%	Recovery/%
1.00	0.97	6.4	97
2.00	1.96	7.5	98
3.00	2.95	5.2	98
4.00	4.07	8.0	101
5.00	4.81	6.9	96

**Table 3:** Immunoassays of CEA and AFP in clinical serum samples by our protocol and the reference method.

	Serum Sample	Reference Method ^a^/ng·mL^−1^		CEA		AFP	
		CEA	AFP	Our Protocol ^b^/ng·mL^−1^	RD/%	Our Protocol ^b^/ng·mL^−1^	RD/%
1	Normal	2.28	11.3	2.19	−3.9	11.1	−1.8
2	Normal	1.72	1.40	1.67	−2.9	1.33	−5.0
3	Normal	1.34	1.31	1.42	6.0	1.39	6.1
4	Pregnant	2.53	14.3	2.62	3.6	13.9	−2.8
5	Lung cancer	5.58	80.3	5.37	−3.8	79.6	−0.9
6	Rectal cancer	34.5	370	33.1	−4.0	369	−0.3
7	Liver cancer	5.02	30.6	5.21	3.8	32.1	4.9

^a^ The reference method was chemiluminescence conducted on an Anthos Lucy 2 semi-automatic analyzer in the hospital; and ^b^ given as the average value of three successive assays.

## Supplementary Materials

The following are available online at http://www.mdpi.com/1424-8220/16/9/1342/s1, Figure S1: *δ* versus volume of 0.1 M HNO_3_ used to dissolve CdS QDs for our protocol (*n* = 3). Conditions: 500-s enrichment; others are the same as in Figure 1 except for varying volume of HNO_3_, Figure S2: Differential pulse ASV responses at a BSA/anti-CEA/GA-CS/SPCE (**a**) and a neighboring bare SPCE (**b**). The electrodes were incubated with 40 fg·mL^−1^ CEA and then Ab_2_-CdS QDs, and the ASV analysis were then performed. Here, only the immunoelectrode showed an ASV peak, while no obvious response was observed at the bare SPCE, Table S1: Immunoassay of CEA in clinical serum samples by our protocol and the hospital method.

## Abbreviations

Ab	Antibody
AFP	*α*-fetoprotein
Ag	Antigen
anti-IgG	Anti-human immunoglobulin G
ASV	Anodic stripping voltammetry
AuNPs	Gold nanoparticles
BSA	Bovine serum albumin
CE	Counter electrode
CEA	Carcinoembryonic antigen
CS	Chitosan
CV	Cyclic voltammetry
EDC	1-ethyl-3-(3-dimethylaminopropyl) carbodiimide
GA	Glutaraldehyde
GCE	Glassy carbon electrode
IgG	Immunoglobulin G
LOD	Limit of detection
LSV	Linear sweep voltammetry
MLAB	Metal-labeled amperometric bioassay
NHS	*N*-hydroxysuccinimide
QDs	Quantum dots
RE	Reference electrode
RD	Relative deviation
RSD	Relative standard deviation
SCE	Saturated calomel electrode
WE	Working electrode
