# Supplementary material for: Sensitive Bioanalysis Based on in-Situ Droplet Anodic Stripping Voltammetric Detection of CdS Quantum Dots Label after Enhanced Cathodic Preconcentration
Source: Sensors (Basel). 2016 Aug 23;16(9):1342. doi: 10.3390/s16091342 (PMC5038621; doi:10.3390/s16091342)
Supplement: Supplementary file 1 [file sensors-16-01342-s001.pdf]

Supplementary Materials: Sensitive Bioanalysis Based on in-Situ Droplet Anodic Stripping Voltammetric Detection of CdS Quantum Dots Label after Enhanced Cathodic Preconcentration

Xiaoli Qin, Linchun Wang and Qingji Xie

Table S1. Immunoassay of CEA in clinical serum samples by our protocol and the hospital.

Method	Serum Sample	Hospital Method [a]/ng·mL^{-1}	Our Protocol [b]/ng·L^{-1}	RD [c]/%
1	Normal	0.89	0.83	−6.7
2	Normal	1.39	1.43	2.9
3	Normal	2.08	2.21	6.3
4	Pregnant	2.28	2.42	6.1
5	Lung cancer	5.58	5.37	−3.8
6	Rectal cancer	34.5	33.1	−4.0
7	Liver cancer	5.04	5.21	3.4

[a] The hospital method was chemiluminescence method conducted on an Anthos Lucy 2 semi-automatic analyzer; [b] Given as the average value of three successive assays; [c] RD: relative deviation.

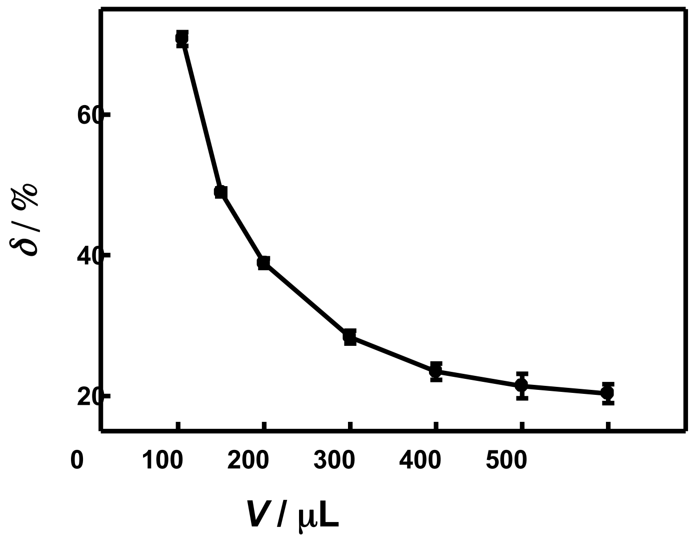

Figure S1. δ versus volume of 0.1 M HNO_3 used to dissolve CdS QDs for our protocol (n = 3). Conditions: 500-s enrichment; others are the same as in Figure 1 except for varying volume of HNO_3.

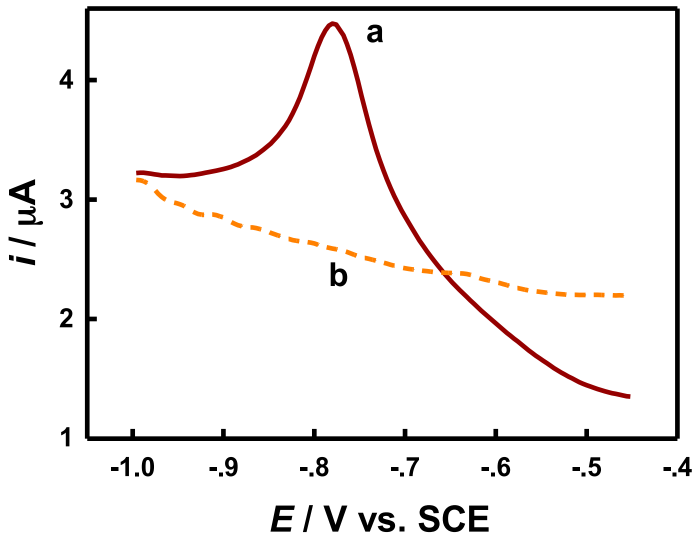

Figure S2. Differential pulse ASV responses at a BSA/anti-CEA/GA-CS/SPCE (**a**) and a neighboring bare SPCE (**b**). The electrodes were incubated with 40 fg·mL^{-1} CEA and then Ab_2-CdS QDs, and the ASV analysis was then performed. Here, only the immunoelectrode showed an ASV peak, while no obvious response was observed at the bare SPCE.
